# Neurogeriatrics—a vision for improved care and research for geriatric patients with predominating neurological disabilities

**DOI:** 10.1007/s00391-020-01734-1

**Published:** 2020-05-19

**Authors:** Andreas H. Jacobs, Kirsten Emmert, Ralf Baron, Thorsten Bartsch, Juergen Bauer, Clemens Becker, Daniela Berg, Philipp Bergmann, Kai Boetzel, Cornelius Bollheimer, Guenther Deuschl, Marija Djukic, Michael Drey, Herbert Durwen, Georg Ebersbach, Morad Elshehabi, Johanna Geritz, Christoph Gisinger, Thomas Guennewig, Bjoern Hauptmann, Hans-Juergen Heppner, Markus A. Hobert, Werner Hofmann, Philipp Huellemann, Klaus Jahn, Jochen Klucken, Roland Kurth, Reinhard Lindner, Paul Lingor, Albert Lukas, Sara Maetzold, Thomas Mokrusch, Brit Mollenhauer, Roland Nau, Annika Plate, Maria Cristina Polidori, Tino Prell, Peter Schellinger, Dominik Spira, Ulrich Stephani, Simone Studt, Claudia Trenkwalder, Heinz L. Unger, Peter Urban, Christine A. F. von Arnim, Tobias Warnecke, Michael Weiss, Andreas Wiedemann, Rainer Wirth, Karsten Witt, Richard Dodel, Walter Maetzler

**Affiliations:** 1 Department for Geriatric Medicine and Neurology, Johanniter Hospital, Bonn and European Institute for Molecular Imaging (EIMI), University of Münster, Münster, Germany; 2 Department of Neurology, University Hospital Schleswig-Holstein Campus Kiel, Kiel University, Arnold-Heller-Str. 3, House D, 24105 Kiel, Germany; 3 Center for Geriatric Medicine, University of Heidelberg and Agaplesion Bethanien Hospital, Heidelberg, Germany; 4 Department of Clinical Gerontology, Robert Bosch Hospital, Stuttgart, Germany; 5 Department of Internal Medicine I, University Hospital Schleswig-Holstein Campus Kiel, Kiel University, Kiel, Germany; 6 Department of Neurology, University Hospital LMU Munich, Munich, Germany; 7 Department of Geriatric Medicine, Medical Faculty, RWTH Aachen University, Aachen, Germany; 8 Department of Neuropathology, University Medical Center Göttingen, Göttingen, Germany; 9 Department of Medicine IV, University Hospital LMU Munich, Munich, Germany; 10 Department of Geriatric Medicine, St. Martinus Hospital Düsseldorf, Düsseldorf, Germany; 11 Hospital for Movement Disorders/Parkinson’s Disease, Beelitz-Heilstätten, Beelitz, Germany; 12 Center for Geriatric Medicine and Geriatric Nursing, Danube University Krems, Krems an der Donau, Austria; 13 Department of Geriatrics and Neurology, Elisabeth Hospital Recklinghausen, Recklinghausen, Germany; 14 Department of Neurology, Segeberger Kliniken, Bad Segeberg, Germany; 15 Department of Geriatrics, University Witten/Herdecke, Schwelm, Germany; 16 Geriatric Center Neumünster and Bad Bramstedt, Friedrich-Ebert-Hospital Neumünster, Neumünster, Germany; 17 Schön Klinik Bad Aibling, Bad Aibling, Germany; 18 Department of Molecular Neurology, University Hospital Erlangen, Friedrich-Alexander University (FAU) Erlangen-Nürnberg, Erlangen, Germany; 19 Neurological Practice Roland Kurth, Kiel, Germany; 20 Institute for Social Work, University of Kassel, Kassel, Germany; 21 Department of Neurology, Technical University of Munich, Munich, Germany; 22 Agaplesion Bethesda Clinic, Competence Centre of Geriatrics and Aging Research, University of Ulm, Ulm, Germany; 23 Department of Neurology and Early Neurological Rehabilitation, MediClin Hedon Klinik Lingen, Lingen, Germany; 24 Paracelsus-Elena-Klinik Kassel, Kassel, Germany; 25 Department of Geriatrics, Protestant Hospital Göttingen-Weende, Göttingen, Germany; 26 Ageing Clinical Research, Dpt. II Internal Medicine, University Hospital of Cologne, and Cologne Cluster of Excellence in Cellular Stress Responses in Aging-associated Diseases, Cologne, Germany; 27 Department of Neurology, Jena University Hospital, Jena, Germany; 28 Departments of Neurology and Neurogeriatry, Johannes Wesling Medical Center Minden, Ruhr University Bochum, Minden, Germany; 29 Department of Endocrinology and Metabolism, Charité—University Medical Center, Berlin, Germany; 30 Department of Neuropediatrics, University Hospital Schleswig-Holstein Campus Kiel, Kiel University, Kiel, Germany; 31 Gerontopsychiatry, Department of Psychiatry and Psychotherapy, University Hospital Schleswig-Holstein Campus Kiel, Kiel University, Kiel, Germany; 32 Clinic of Neurosurgery, University Medical Center Göttingen, Göttingen, Germany; 33 Department of Geriatrics and Early Rehabilitation, Evangelical Hospital Kalk Cologne, Cologne, Germany; 34 Department of Neurology, Asklepios Klinik Barmbek, Hamburg, Germany; 35 Department of Geriatrics, University Medical Center Göttingen, Göttingen, Germany; 36 Department of Neurology, University of Münster, Münster, Germany; 37 Clinic for Neurology and Clinical Neurophysiology, Schön Klinik Neustadt, Neustadt, Germany; 38 Department of Urology, Evangelical Hospital Witten, Witten, Germany; 39 Department of Geriatric Medicine, Marien Hospital Herne, University Hospital Ruhr University Bochum, Bochum, Germany; 40 Department of Neurology, Carl von Ossietzky University Oldenburg, Oldenburg, Germany; 41 Chair of Geriatrics, University Hospital Essen and Geriatriezentrum Haus Berge, Contilia Group, Essen, Germany; 42 Department of Therapeutic Sciences, MSH Medical School Hamburg, Hamburg, Germany; 43 Helios Clinic Schwelm, Schwelm, Germany; 44 German Center for Vertigo and Balance Disorders (DSGZ), Ludwig-Maximilians-University of Munich, Munich, Germany; 45 Research Group Digital Health Pathways, Fraunhofer IIS, Erlangen, Germany; 46 Department of Neurology, University Medical Center Göttingen, Göttingen, Germany; 47 Malteser Hospital Bonn, Geriatric Centre, Academic Teaching Hospital, University of Bonn, Bonn, Germany; 48 Paracelsus-Elena Klinik, Kassel, Germany; 49 Department of Geriatrics, Witten-Herdecke University, Witten, Germany

**Keywords:** Centers for Aging Medicine, Neurogeriatric assessment, Interdisciplinarity, Multidisciplinary team, Neurology, Cognitive decline, Movement disorder, Zentren für Altersmedizin, Neurogeriatrische Untersuchung, Interdisziplinarität, Multidisziplinäres Team, Neurologie, Kognitive Störung, Bewegungsstörung

## Abstract

Geriatric medicine is a rapidly evolving field that addresses diagnostic, therapeutic and care aspects of older adults. Some disabilities and disorders affecting cognition (e.g. dementia), motor function (e.g. stroke, Parkinson’s disease, neuropathies), mood (e.g. depression), behavior (e.g. delirium) and chronic pain disorders are particularly frequent in old subjects. As knowledge about these age-associated conditions and disabilities is steadily increasing, the integral implementation of neurogeriatric knowledge in geriatric medicine and specific neurogeriatric research is essential to develop the field. This article discusses how neurological know-how could be integrated in academic geriatric medicine to improve care of neurogeriatric patients, to foster neurogeriatric research and training concepts and to provide innovative care concepts for geriatric patients with predominant neurological conditions and disabilities.

## Abstract (de)

Die Geriatrie ist ein sich rasch entwickelnder Fachbereich, der sich mit der Diagnose, Therapie und Pflege von älteren Erwachsenen befasst. Einige Erkrankungen und Störungen, die Kognition (z. B. Demenz), Motorik (z. B. Schlaganfall, ParkinsonSyndrome, Neuropathien), Emotionen (z. B. Depression), Verhalten (z. B. Delirium) und chronische Schmerzerkrankungen betreffen, sind bei alten Menschen besonders häufig. Da unser Wissen über diese altersbedingten Erkrankungen stetig zunimmt, ist die Umsetzung von neurogeriatrischem Wissen in der geriatrischen Medizin und die spezifische neurogeriatrische Forschung für die Entwicklung des Fachgebietes unerlässlich. Dieser Artikel stellt Szenarien vor, wie neurologisches Wissen in die akademische Geriatrie integriert werden kann, um die Versorgung neurogeriatrischer Patienten zu verbessern, neurogeriatrische Forschungs- und Ausbildungskonzepte zu fördern und innovative Versorgungskonzepte für geriatrische Patienten mit führend neurologischen Erkrankungen und Störungen umzusetzen.

## The need for specialized care and research in geriatric medicine

Geriatric medicine, dedicated to providing care for older adults, is an innovative medical specialty of considerable importance for several reasons. Firstly, a considerable increase in older patients is expected worldwide, and geriatric medicine will become even more important due to this demographic change. Secondly, geriatric medicine provides important value-based healthcare to the patient [16]. Thirdly, geriatric medicine is strongly oriented towards the International Classification of Function and Disability (ICF model) introduced by the World Health Organization (WHO) in 2001 [31]. Clinical geriatric assessment and management are comprehensive as well as function and person-oriented. With these strategies geriatric medicine played a pioneering role for the implementation of person-centered care from its very beginning, comparable to palliative care, psychosomatics and psychiatry. Geriatrics has been fostering person-centered and goal-oriented care in policy and many other medical disciplines, complementing the already far-developed organ and disease-oriented treatments provided by almost all other medical specialties.

With the increasing recognition of the importance of geriatric medicine, it has also gained a strong research profile. Fundamental conceptual definitions, descriptions and investigations of multifactorial geriatric syndromes, such as frailty and sarcopenia, falls and immobility, impaired cognition, depression and delirium are some examples of conditions where geriatricians are the accepted experts in the complex decision-making for persons in the context of multimorbidity (i.e., multiple chronic conditions) [27]; however, research activities in the field did not develop as rapidly as the clinical field leading to a translational research and training gap, and geriatric medicine is still underrepresented at academic institutions in Germany. This may be at least in part due to the fact that the field of geriatrics ranks as a subspecialty which can be acquired by additional training after having obtained full board certification in subjects such as internal medicine, neurology or psychiatry. This issue has been addressed by the Robert Bosch Foundation from 2002 to 2015, when it supported advanced training for more than 50 young medical professionals in German-speaking countries and founded full professorships for geriatrics at several universities including the RWTH in Aachen, the Universities of Heidelberg and Göttingen. In addition, the German Federal Ministry of Education and Research (BMBF) launched a call to found new chairs and fund junior research groups in the field of gerontology and geriatrics in 2016. These positions will be funded from 2019 to 2027 with up to 3.5 million €; however, academic geriatric medicine faces relevant challenges, e.g. to ensure high quality research approaches and output as well as recruitment and training of young geriatricians in the area of diagnosis and care of older adults [27]. It is therefore necessary that, at least in the university environment, geriatric units are able to develop further into highly specialized academic centers, where, in addition to standard interdisciplinary geriatric care, high-level clinical research and training will be possible to drive the entire field forward. It should be pointed out that in general in Germany the primary clinical care of patients is not part of academic centers but is mostly organized in regular hospitals, which also impedes scientific research.

Currently, many geriatricians define the field as a general medical specialty that takes over old patients from other medical fields when functional impairment (e.g. motor function, cognition), measured as impairment of activities of daily living (ADL) and independence, is predominant. According to the European Union of Medical Specialists (UEMS) “Geriatric Medicine is a specialty of medicine concerned with physical, mental, functional and social conditions in acute, chronic, rehabilitative, preventive, and end of life care in older patients. This group of patients are considered to have a high degree of frailty and active multiple pathology, requiring a holistic approach. Diseases may present differently in old age, are often very difficult to diagnose, the response to treatment is often delayed and there is frequently a need for social support. Geriatric Medicine therefore exceeds organ orientated medicine offering additional therapy in a multidisciplinary team setting, the main aim of which is to optimise the functional status of the older person and improve the quality of life and autonomy” (https://uemsgeriatricmedicine.org/www/land/definition/english.asp). The dynamic increase of knowledge within medical technologies and skills within and across medical specialties requires that geriatric medicine provides the highest level of state of the art care to any old patient, independent of the leading disability, and designs adequate research strategies to provide evidence-based outcome data (e.g. functional improvement, reduction of falls).

**Fig. 1:**
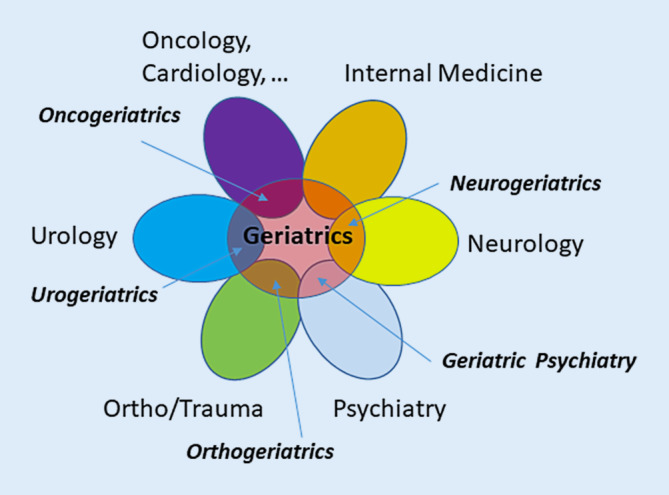
The field of geriatrics with its high expertise in interdisciplinary teamwork can fuel into the mostly organ-oriented medical fields for the formation of various dedicated specialties (e.g. oncogeriatrics, urogeriatrics, orthogeriatrics, geriatric psychiatry, neurogeriatrics). This may lead to the formation of centres for aging medicine

The rapid technological advances of modern medicine (e.g. genome sequencing/editing, personalized targeted medicine, multimodal molecular imaging, robotics, artificial intelligence, precision medicine) have reached the field of geriatric medicine. An example is the geriatric assessment of cancer patients at old age to estimate the risk-benefit ratio of a molecular targeted therapy [14]. As originally proposed by Warren [11] it is argued here that these clinical and technological advances are best implemented in specialized multidisciplinary (multiprofessional, multispecialty) teams. The improved outcome of care by dedicated multidisciplinary teams has been shown, e.g. for (i) old patients with functional impairments and multimorbidity within dedicated geriatric units [2], (ii) for patients with stroke or heart attack within stroke or chest pain units [9], (iii) for the management of old patients within the emergency department [15] and (iv) for older patients with trauma and fractures within orthogeriatric units [18, 19]. Only within these specialized units is knowledge and know-how available to optimally prevent deterioration/complications and to improve the condition as best as possible (Fig. 1).

Therefore, the field of geriatrics currently faces the pressure to integrate as many disciplines into its multiprofessional teams as possible and to further specialize; health insurances already call for specialized orthogeriatric treatment in the field of hip fractures (Weissbuch Alterstraumatologie; www.dggeriatrie.de/presse/pressemeldungen). These structures also fit with initiatives coming from policy makers, e.g. from the German Science Council [4].

## Neurogeriatric care and research: an example for specialized geriatrics

**Fig. 2:**
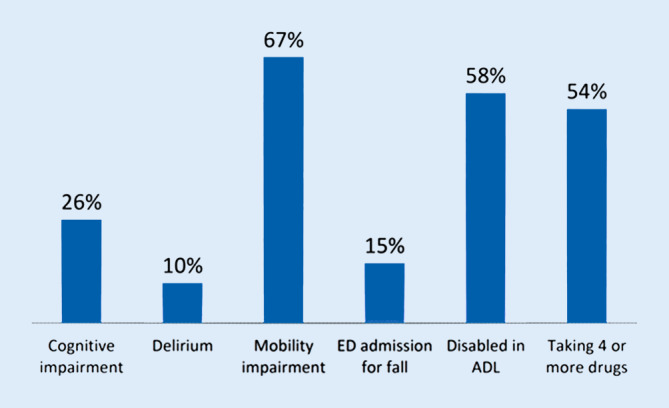
Prevalence of health disabilities in patients ≥75 years old referred to the general emergency room at the University Hospital Bern from 2009–2012. From [6] with courtesy. *ED* emergency department, *ADL* activity of daily living

A survey of the prevalence of functional limitations in adults aged >75 years referred to the general emergency room of a university hospital showed that two thirds had mobility limitations, 26% of these patients had cognitive deficits, 1 in 7 was referred due to a fall, and 1 in 10 suffered from delirium (Fig. 2; [21]).

These data indicate the high prevalence of geriatric disabilities in adults aged >75 years where the integration of neurogeriatric expertise would be beneficial, if not essential, for the routine clinical management. They are either caused, at least to a relevant extent, by a neurological deficit, e.g. mobility impairment and falls due to vertigo, stroke/small vessel disease, Parkinson’s disease (PD), polyneuropathy [3, 6] or need neurogeriatric expertise to differentiate from other causes (cardiac, musculoskeletal) or to adequately address diagnosis and treatment (e.g. cognitive impairment and delirium [1]). Disturbances of motor and cognitive functions occur regularly in age-related neurological diseases, such as cerebrovascular and small vessel diseases, Alzheimer’s disease (AD) and PD [3, 6] and in systemic conditions, such as (i) inflammation (due to an altered blood-brain barrier with microglia activation), (ii) cardiovascular dysfunction or due to (iii) drug interactions and side effects including interference with neurotransmitter systems. For example, the interaction of frailty with both the occurrence and severity of amyloid deposition or small vessel disease in the brain and the manifestation of dementia has recently been demonstrated and supports the idea of a central regulatory role of brain damage with aging [29]. Taken together, dysregulation of the central nervous system (CNS) and peripheral nervous system (PNS) networks by direct or indirect damage may lead to functional disturbances observed in geriatric patients. Therefore, the integration of neurological/neurogeriatric expertise into the geriatric team in any type of setting seems to be essential.

In 2017 more than 40 scientifically active specialists in geriatrics and neurology met in Kiel, Germany, to further conceptualize the field of neurogeriatrics. The consortium agreed on the urgent need for an operational definition [12] and for more specific research in this area. Neurogeriatrics was defined as a medical area dedicated to geriatric patients with predominating neurological disabilities. The consortium also introduced neurogeriatric task forces working on the topics of mobility, the three Ds (dementia, delirium, depression)^1^, and dysphagia/malnutrition, with the aim to define specific academically driven multicentric study protocols for translational research, the standardized assessment and evaluation of care for geriatric patients. Examples for research topics where neurogeriatrics should be involved are presented in the following section.

Mobility impairment has repeatedly been shown to be associated with cognitive deficits and falls, and research on gait, balance, visual and cognitive issues as well as on effective modes of intervention is already well[3]. Future neurogeriatric research in this field should answer the questions (i) which patient cohorts/disease subtypes benefit most, and which may even suffer damage with the identical intervention; (ii) which specific gait, balance, visual and cognitive parameters are responsible for the effects. In PD, a recent study showed that gait variability is strongly associated with falls and that stimulation of the cholinergic system may improve both [7]. It was also shown that a motor-motor (but not a motor-cognitive) dual tasking deficit is a strong predictor for falls in early to moderate PD [6]. These findings will be the basis for more effective interventions and preventions in (geriatric) PD cohorts with falls and may serve to generate new research hypotheses in non-PD geriatric patients suffering from falls. Interestingly, fall intervention has been demonstrated to be beneficial in early to moderate but not to advanced stages of PD [24], indicating that even in neurogeriatric cohorts, subcohorts may exist that show different treatment response. To the best of our knowledge, no data exist at the present about a cost-benefit ratio in other geriatric conditions involving falls.

Associations of visual impairment and especially eye movement deficits with falls in PD and atypical Parkinsonian disorders are currently being investigated in a prospective study [25], suggesting that an impairment of the vestibulo-ocular reflex (VOR) suppression is strongly associated with falls in some but not all age-related diseases with neurological symptoms. Disturbances in VOR are associated with diseases at advanced age, such as bilateral vestibulopathy, cerebellar type of multiple systems atrophy (MSAc), and CANVAS syndrome (cerebellar ataxia, peripheral neuropathy, vestibular areflexia syndrome). These conditions are frequent in a general geriatric population, and physicians have to learn how to establish proper training methods to prevent falls in these conditions.

**Fig. 3:**
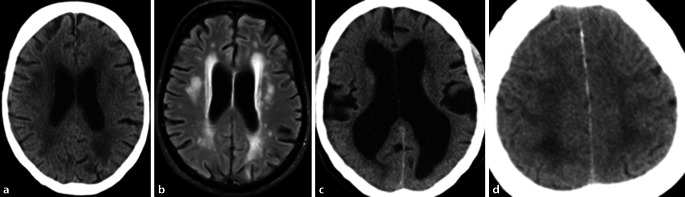
Axial sections showning signs of subcortical hypodensities (CCT, **a**) or hyperintensities (MRI, **b**) as manifestation of atherosclerotic encephalopathy (SAE) leading to impairment of motor function and cognition in a 78-year-old patient with diabetes mellitus and hypertension. Signs of normal pressure hydrocephalus (NPH) with dilatation of ventricles (**c**) and tight sulci in the high frontoparietal regions (**d**) as a typical geriatric syndrome with gait disturbances, falls, impaired cognition and urinary incontinence

Small vessel disease leading to subcortical atherosclerotic encephalopathy depicted as white matter hyperintensities (WMH) is regularly associated with cognitive disturbances, gait disorders and falls (Fig. 3a, b). The WMH are probably the most frequent cause of functional impairment within the central motor and cognitive domain. A recent study showed that specific mobile device-derived physical activity parameters can predict future falls in people with dementia [22], which motivates further studies that differentiate between dementia subtypes under consideration of multimorbidity. Another study demonstrated that physical training improves motor performance in patients with dementia (without further subtyping) [5, 23]. Here, the next step is to delineate whether this effect can be observed in different dementia subtypes (with and without WMH) and whether this is a subtype-specific effect. The common age-related disease, normal pressure hydrocephalus (NPH, Fig. 3c, d) with the diagnostic triad of gait disturbance, cognitive impairment and urinary incontinence, may serve as a prototype neurogeriatric syndrome in this respect.

Besides the fields of mobility and cognition with overlap to delirium and depression also other geriatric syndromes, namely oropharyngeal dysphagia and malnutrition need special attention and are research topics where neurogeriatrics could lead to improving the understanding of these conditions. Malnutrition is common and has been shown to contribute to a lower quality of life, an increase in the length of stay in hospital and increased rates of readmission, and higher morbidity and mortality in older people [20, 26]. The genesis of malnutrition is multifactorial. In addition to age-related physiological changes which favor loss of appetite and/or decreased food intake in late life (anorexia of aging) [10] malnutrition is frequently associated with oropharyngeal dysphagia [30], cognitive decline, gait disorders, movement disorders (i.e. PD) and inflammatory processes. In particular, disease-specific aspects and predictors of malnutrition in neurogeriatric patients are important to understand in order to perform an effective screening and to start interventions at the right time in older patients with neurological disorders [17].

Further examples for the utility of, and the need for improvement of neurogeriatric knowledge for an understanding of common geriatric conditions were presented in a special issue on neurogeriatrics (Z Gerontol Geriatr July 2019), including cognitive and mobility deficits, dysphagia and functional movement disorders [1, 8, 13, 30].

## Multidisciplinary geriatrics: neurogeriatrics as an example

Geriatric care on an individual patient level must be based on multiprofessional geriatric teams. The authors argue here that it needs, in addition, medical knowledge and expertise provided at the point of care coming from the interaction of different medical specialties with geriatric expertise, including internal medicine, neurology, psychiatry, pain medicine and orthopedics. The advantage of this multidisciplinary approach has already been demonstrated in numerous studies and trials, and has led to the implementation of dedicated care units with leading geriatric involvement in routine medical care ([2, 9]; Fig. 1). A structured evaluation of the effects, effectiveness and efficiency of the management of geriatric patients with leading neurological symptoms and functional deficits in specialized neurogeriatric care units is required in the future.

In addition to the clinical multidisciplinary or multispecialty geriatric approach a strong interaction with basic age-related research is necessary. General geriatric and specialized (e.g. neurogeriatrics) clinical and research groups should incorporate basic science-related work or at least collaborate with basic science research groups to investigate mechanisms and markers associated with aging and age-associated diseases and functional disturbances. This can identify novel research gaps and fuel research in translational science. Examples for the field of neurogeriatrics include the microbiome changes during aging and its influence on neurodegeneration, age and systemic disease-associated alterations of eye structures, CNS changes in diabetes, and mobility assessment in the usual environments and delirium prevention of old patients undergoing cardiac surgery.

The dedicated aims of research-oriented (neuro)geriatric networks and infrastructures proposed here are (i) to foster clinical teams to further develop geriatric assessment and management strategies to be disease and specialty-adapted; (ii) to build core teams of highly skilled academic experts with profound knowledge on the complex etiology and interconnectivity of diseases in older persons, on the state of the art of comprehensive, function and person-centered assessments as well as on care strategies for older adults with complex conditions; (iii) to support collaborations with disciplines at the direct interfaces of geriatric medicine (e.g., omics, biobanking, epidemiology, prevention, techniques, ethics, systems biology, statistics) to continuously improve our knowledge of diseases and functional disturbances in older persons with a focus on neurological symptoms; and (iv) to provide realistic and appealing career pipelines in the field of (neuro)geriatric medicine for young clinicians and scientists of all relevant medical disciplines. This must include strong initiatives for the foundation and continuation of geriatric chairs and professorships at universities that have the opportunity to develop high-level research at the boundaries of geriatrics and other specialties.

We anticipate that the initiatives described will influence the organization, structure and development of geriatric units in other academic institutions and also in the nonacademic area. Eventually, these developments may serve as a template and building brick for the formation of centers for aging medicine bringing together several specific geriatrics units as described in Fig. 1 together with basic aging research activities to allow knowledge gain and improved interdisciplinary care of old patients.

## Conclusion

Neurological expertise is indispensable within a multiprofessional geriatric team. The implementation of neurogeriatrics in the academic setting will help (i) to create dedicated research infrastructures with interdisciplinary access to patients, data, and biomaterial to advance medical problem solving in geriatric patients with leading neurological symptoms and functional deficits, (ii) to initiate and support basic science-oriented research, (iii) to attract and fascinate young medical professionals for the increasing need to provide highly innovative and specialized geriatric medicine and care to our aging society and (iv) to collect effectiveness and efficiency data about geriatric care, further stimulating adaptive processes of geriatric medicine in general.

## Footnotes

1 These conditions have been, and still are, often researched and managed by neuropsychiatrists, reflecting more than “just a bridge between neurology and psychiatry” [28]. This situation is comparable to neurogeriatrics.
